# Diagnosis of visceral and cutaneous leishmaniasis using loop-mediated isothermal amplification (LAMP) protocols: a systematic review and meta-analysis

**DOI:** 10.1186/s13071-021-05133-2

**Published:** 2022-01-24

**Authors:** Astrid Christine Erber, Peter Julian Sandler, Daniel Moreira de Avelar, Ines Swoboda, Gláucia Cota, Julia Walochnik

**Affiliations:** 1 Department of Epidemiology, Center for Public Health, Medical University of Vienna, Kinderspitalgasse 15, 1st floor, 1090 Vienna, Austria; 2 Nuffield Department of Medicine, Centre for Tropical Medicine and Global Health, University of Oxford, New Richards Building, Old Road Campus, Roosevelt Drive, Oxford, OX3 7LG UK; 3 Department of Applied Life Sciences, FH Campus Wien University of Applied Sciences, Helmut-Qualtinger Gasse 2, 1030 Vienna, Austria; 4 Pesquisa Clínica e Políticas Públicas em Doenças Infecciosas e Parasitárias, Instituto René Rachou—Fundação Oswaldo Cruz, Fiocruz, Belo Horizonte, Minas Gerais Brazil; 5 Institute of Specific Prophylaxis and Tropical Medicine, Medical University of Vienna, Kinderspitalgasse 15, 1090 Vienna, Austria

**Keywords:** Leishmaniasis, Cutaneous leishmaniasis, Visceral leishmaniasis, Loop-mediated isothermal amplification, In vitro diagnostics, Neglected tropical disease, Meta-analysis

## Abstract

Sensitive, reliable and fast diagnostic tools that are applicable in low-resource settings, at the point of care (PoC), are seen as crucial in the fight against visceral leishmaniasis (VL) and cutaneous leishmaniasis (CL). Addressing the need for a PoC test, several diagnostic tests, including serological and molecular methods, have been developed and evaluated in the past. One promising molecular method, already implemented for diagnosis of a range of diseases, is the loop-mediated isothermal amplification (LAMP) protocol. In this systematic review and meta-analysis, using a comprehensive search strategy, we focus on studies evaluating the performance of LAMP for the diagnosis of leishmaniasis in humans and other mammals such as dogs, compared with microscopy and/or any other molecular diagnostic method. A meta-analysis, pooling sensitivity and specificity rates and calculating areas under the curve (AUCs) in summary receiver operating characteristic (SROC) plots, was conducted on datasets extracted from studies, grouped by clinical condition and sample type. We found high sensitivity and specificity for LAMP when compared with microscopy and PCR using blood samples, with pooled estimate values of > 90% for all subgroups, corresponding to calculated AUC values > 0.96, except for LAMP compared to microscopy for diagnosis of CL. However, only a limited number of studies were truly comparable. Most of the observed heterogeneity is likely based on true differences between the studies rather than sampling error only. Due to simple readout methods and low laboratory equipment requirements for sample preparation compared to other molecular methods, LAMP is a promising candidate for a molecular (near-)PoC diagnostic method for VL and CL.

**Graphical Abstract:**

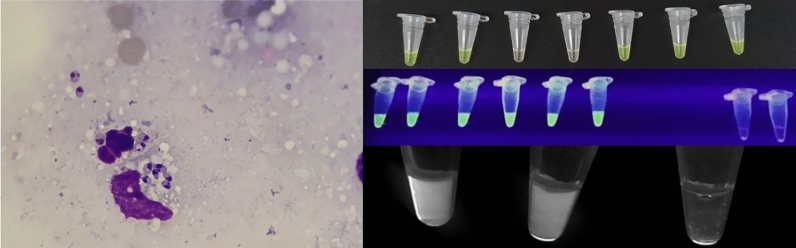

**Supplementary Information:**

The online version contains supplementary material available at 10.1186/s13071-021-05133-2.

## Background

Leishmaniasis is a vector-borne disease caused by protozoan parasites of the genus *Leishmania* [1] and transmitted by the females of phlebotomine sand flies [2, 3]. Factors such as proximity of animal reservoirs in the current model of peri-urban transmission, different susceptibilities of human populations and the environmental impact on vector distribution result in a complex interplay [4, 5]. There are various clinical manifestations, but a widely used classification differentiates between visceral leishmaniasis (VL), which is fatal if left untreated, cutaneous leishmaniasis (CL), mucocutaneous leishmaniasis (MCL), and a possible concurrent or late-term complication of VL which is called post kala-azar dermal leishmaniasis (PKDL) [6, 7]. Globally, there are about 12 million patients suffering from leishmaniasis, with more than 350 million people at risk in over 80 countries [8]. The World Health Organization (WHO) estimates that around 0.7–1.0 million cases occur annually, 50,000 to 90,000 of which are VL cases and 0.6–1.0 million CL cases [9]. Based on data from the Global Health Observatory data repository for 2018, 17,000 VL [10] and 250,000 CL [11] cases were reported to WHO by 53 countries. However, official numbers may be an underestimation for different reasons such as VL-related deaths outside of health care facilities [12]. In addition, not all endemic countries reported data to WHO in 2018. Around 90% of VL cases occur in six countries: Bangladesh, Brazil, Ethiopia, India, South Sudan and Sudan. CL is distributed globally; the most affected countries are Afghanistan, Pakistan, Iran, Syria, Saudi Arabia, Algeria, Brazil, Colombia and Peru, with recent epidemics in Afghanistan and Syria [11, 13]. The majority of VL infections are caused by *Leishmania donovani* and *Leishmania infantum* [14]. Several *Leishmania* species can cause CL; the most common causes of the infection are the species *Leishmania major*, *Leishmania tropica*, *L. infantum* (Mediterranean Basin, the Middle East, the Horn of Africa, Indian subcontinent), *Leishmania aethiopica* (in Ethiopia and Kenya), *Leishmania braziliensis*, *Leishmania guyanensis* (South America), and *Leishmania mexicana* (Mexico) [15–18]*.* In some regions of the Southern Hemisphere, especially in South America, the areas endemic for *Leishmania* have been expanding in the recent past [19, 20]. In addition, due to climatic change, more habitats will become suitable for phlebotomine sand flies, resulting in a possible expansion of their geographic ranges and an establishment of endemic *Leishmania* transmission in more extreme latitudes throughout the world [19–22].

Clinical symptoms of VL include fever, anaemia, leukopenia, hepatosplenomegaly, weight loss and diarrhoea. Most VL infections remain asymptomatic, but long incubation periods of up to 8 months are not uncommon, and symptomatic infections are often fatal if left untreated [6, 23]. The symptoms are similar to other diseases such as malaria and enteric fever, and a laboratory diagnosis is required for accurate diagnosis [24]. Treatment recommendations for VL differ between regions but commonly used drugs are (liposomal) amphotericin B and pentavalent antimonials, both administered intravenously, or miltefosine, used orally [6, 25]. Therapeutic studies in the past focused mainly on monotherapy and the combination of existing drugs, but the Drugs for Neglected Diseases *initiative* (DND*i*) has identified several candidates which might lead to innovative treatments for VL [26].

The majority of CL cases manifest as chronic and normally painless skin lesions. These may heal spontaneously in response to development of cell-mediated immunity if untreated, although in most cases this process takes several months and up to years [27], with typically a low percentage of self-healing lesions for New World CL [28, 29]. Treatment of CL may include systemic therapy or local therapy such as heat or cryotherapy, topical creams (e.g. paromomycin) or intralesional injections of pentavalent antimonial derivatives [30]. Lesions may leave disfiguring scars, possibly leading to stigmatization of recovered patients, having a long-term negative impact on psychological, social and economic well-being [31, 32]. In contrast to CL, MCL is potentially life-threatening if untreated. Ninety percent of MCL cases have a scar from a prior CL episode; depending on host cell-mediated immunity and parasite virulence, clinical progression to the mucosa may take place. Symptoms of an infection are progressive destruction of the oronasopharyngeal mucosa and cartilaginous facial and upper airway structures [33]. The ratio of MCL to CL infections is low, and disease progression may be strongly dependent on the infecting species and possibly also on their infection with *Leishmania* RNA viruses [34, 35].

PKDL mostly occurs in eastern Africa and on the Indian subcontinent and is associated with a previous VL infection in most cases. It is manifested by mostly self-healing lesions which are only aesthetic problems in most infected individuals but are infectious to phlebotomine sand flies, possibly over decades [6].

There are numerous different diagnostic test methods available for leishmaniasis, which can be divided into non-DNA-based and DNA-based methods [36]. Among the non-DNA-based are serological methods detecting antibodies or antigens (such as proteins), and microscopic methods, which have long been regarded as the gold standard for VL and CL diagnosis [37]. For VL diagnosis, the acquisition of tissue samples for microscopic methods is highly invasive, as spleen, lymph node or bone marrow aspirates are needed [38]. For CL diagnosis, the sensitivity of microscopy is only moderate [6, 16].

Serological tests are less invasive and can be used in a near-PoC setting to support clinical VL diagnosis, as they generally have high sensitivity and low costs, and results can be determined in the field [39–41], but tests based on detection of antibodies largely cannot distinguish between current and past infections [42]. Sensitivity is lower in immunocompromised individuals such as HIV-co-infected patients and in very young children [24, 43–45]. Furthermore, cross-reactivities are possible [46–48]. Different from VL and partly also MCL, serological methods have low sensitivity in CL [16], as this disease usually only leads to a local immune response [49]. Rapid diagnostic tests (RDTs) based on the detection of the rK39 antigen are widely used and reliable for diagnosis of VL [50].

DNA-based test methods usually have high sensitivity and specificity, but require laboratory equipment such as a thermocycler and cold chain-kept reagents and are therefore difficult to implement in point-of-care (PoC) or near-PoC settings [51, 52]. In addition, laboratory staff need to be trained appropriately and there are concerns regarding the lack of standardization and quality control of molecular assays [53]. However, they can also be applied to immunocompromised patients [24] and, importantly, they do not require invasive sampling methods and can be performed with peripheral blood (VL) or lesion swab sampling (CL) [53].

Polymerase chain reaction (PCR) and quantitative real-time PCR (qPCR) are among the most widely used DNA-based test methods [5]. Nested PCR (LnPCR) increases the sensitivity in samples with low parasite density but is prone to contamination. Multiplex assays can detect several species (or species groups) at the same time but are also more expensive [54].

Another promising molecular method for diagnosis of VL and CL is the loop-mediated isothermal amplification method (LAMP). LAMP uses a polymerase and typically four primers to amplify six target regions under isothermal conditions with high specificity. One of the inner forward and backward primers contains a complementary sequence which leads initially to a loop formation and in later amplification circles to dumbbell structures, forming continuously growing concatemers [55, 56]. LAMP has high specificity because amplification only occurs if all six target regions are correctly recognized by the primers [57]. Since a large number of amplicons are produced and only a small quantity of sample is needed for successful amplification via LAMP, contamination of the workplace by amplicons of previous samples has been identified as a potential risk resulting in false-positive results [58–60]. This risk can be reduced by using closed tubes which do not need to be opened to evaluate the result [61–64].

Several methods for visual evaluation of amplification results have been developed. Pyrophosphate ions, which are reaction by-products, form a white precipitate with magnesium of the reaction buffer [65], and the addition of manganous ions and calcein leads to a visible colour change, enabling simple visual detection of positive samples without further equipment [66].

SYBR Green, which is a DNA-binding dye that intercalates non-specifically into double-stranded DNA (dsDNA), can also be added to the tube initially blocked by a heat-sensitive capsule, as direct addition inhibits the amplification reaction [62, 64, 67].

LAMP has been used in the diagnosis of a variety of diseases and detection of a whole spectrum of different pathogens in both humans and animals [68]. LAMP has been established for various human pathogens, including *Leishmania* spp. [69], *Trypanosoma brucei gambiense* (human African trypanosomiasis) [70], *Plasmodium falciparum* (malaria) [71], *Burkholderia pseudomallei* (melioidosis) [72], *Mycobacterium tuberculosis* (tuberculosis) [73], *Mycobacterium avium* subsp. *paratuberculosis* (MAP, Johne's disease) [74] and various *Staphylococcus* strains (food-borne infections) [75], among others. LAMP has also been used in combination with a reverse transcriptase enzyme (RT-LAMP) in order to amplify target RNA, making it a possible tool for detection of RNA viruses such as the Newcastle disease virus or SARS-CoV-2 (2019-nCoV) [76, 77]. RT-LAMP has been used to detect hepatitis B virus (hepatitis B) [78], H5N1 highly pathogenic avian influenza (HPAI, avian influenza) [79] and classical swine fever virus (CSFV, swine fever) [80].

To assess the performance of LAMP for CL and VL diagnoses, we conducted a systematic literature review, extracted data from eligible studies, and performed a qualitative and quantitative analysis, with a meta-analysis of selected datasets, to evaluate diagnostic test parameters compared to the well-established and commonly used reference standards microscopy and PCR-based methods (PCR, qPCR, LnPCR).

## Methods

### Literature review protocol preparation

The review protocol was registered in the International Prospective Register of Systematic Reviews (CRD42020150035) and can be accessed at https://www.crd.york.ac.uk/PROSPERO/display_record.php?ID=CRD42020150035. Recommendations of the Cochrane Handbook for Systematic Reviews of Diagnostic Test Accuracy [81] and of the Preferred Reporting Items for Systematic Reviews and Meta-Analyses (PRISMA) statement [82, 83] were followed.

### Data sources and search strategy

Structured searches were conducted by two reviewers on the PubMed and PubMed Central, Scopus, Web of Science, Cochrane Library, Embase, Epistemonikos and Global Index Medicus databases, using a comprehensive list of key terms including leishmania* AND (LAMP OR loop-mediated OR (isothermal AND amplification) but adapted to each database. Serological test methods were considered out of scope for the search strategy and the review overall, as they do not necessarily correlate with an active infection. A detailed description of the search strategy and search dates is available as supplementary information (see Additional file 1: Text S1). The initial search was complemented by a manual search of reference lists from retrieved articles and by citation tracking of review articles. If a study reported diagnostic performance values (e.g. specificity, sensitivity) but contained no individual sample data or information allowing completion of a 2 × 2 contingency table, further information was requested by mail from the corresponding and/or first author. If no further information was acquired, the respective study was included in the qualitative synthesis but not in the statistical analyses or in the meta-analysis. The literature search was conducted in July 2019 and repeated in July 2020 to include studies published up to the end of June 2020.

### Inclusion and exclusion criteria

As inclusion criteria, studies were included if results for LAMP assays for diagnosis of leishmaniasis in clinical samples from humans or animals, with confirmation by microscopy, culture or molecular tests, were reported. No restrictions were made with respect to the publication language, date of publication or study design (consecutive or case–control) or data collection (prospective or retrospective).

As exclusion criteria, studies were excluded in the case of lack of data regarding individual results reported, reference standard used or sample type. In addition, reviews and commentaries were excluded but references were analysed regarding potential further studies meeting the inclusion criteria.

### Selection process

Deduplication of publications found in several databases was done manually and using Zotero 5.0.60 [84]/5.0.84 [85]. After removal of duplicates, each publication had its title and abstract reviewed based on the inclusion and exclusion criteria in a blinded manner by two independent reviewers, using Rayyan [86]. After unblinding, discrepancies were resolved by discussion. In case an abstract did not contain enough information for rejection, the publication was automatically included for the full-text screening. Subsequently, the selected publications were read in full independently by both reviewers, either to confirm their eligibility and to extract the data or to exclude, again after unblinding and discussion with the second independent reviewer.

### Data extraction

Data extraction was conducted by one reviewer and verified by a second reviewer based on a sample set of the included studies. We extracted data from primary studies to complete the four cell values of a diagnostic 2 × 2 table: true positives, false positives, true negatives, and false negatives. In addition, the following information was recorded: infecting species, sample type, reference test, LAMP target, country of patient’s origin, DNA extraction method, readout method of the LAMP and study design (consecutive or case–control).

### Study quality assessment

The quality of included studies and risk of bias and applicability was assessed based on the QUADAS-2 tool [87].

### Data synthesis

The accuracy measurements of interest for LAMP were sensitivity and specificity, which are defined as follows: sensitivity (S)—probability of a positive test in diseased individuals; specificity (E)—probability of a negative test in non-diseased individuals. In order to calculate S and E values for LAMP, we cross-tabulated each result against each one reference standard (microscopy and/or another molecular diagnostic method besides LAMP), stratified by each clinical condition (CL, VL or PKDL) and biological specimen used. Thus, for the same study, more than one analysis was possible: in general, each panel of samples extracted from a single study, tested with LAMP using the same sample type against the same reference standard test, was called “dataset”. For Schallig et al. [52] and Vink et al. [88], two different datasets were created depending on the country where the panel of samples were analysed (see Additional file 2: Table S1, comments).

### Analysis

Descriptive statistics as calculation of mean, median and test for normal distribution (Shapiro–Wilk) were also calculated in R version 3.6.2 [89]. The accuracy measurements were calculated using R and the epiR package version 1.0.10 [90]. For the subsequent meta-analysis, we were interested whether including studies with a sample size below 10 would introduce a bias and should be excluded, in line with previous publications [28, 91]. We therefore calculated Spearman’s rank correlation coefficient, including a 95% confidence interval (CI), in order to analyse the possible correlation between sample size and S or E using R. Forest plots showing S and E values for all datasets including a 95% CI were created using RevMan 5.3 [92].

Subgroup 1 (“VL Microscopy LAMP: Blood” consisting of datasets 1, 8, 23, 38, 42, 44, 47, 59, 64), subgroup 2 (“VL PCR LAMP: Blood” consisting of datasets 11, 26, 30, 33, 35, 36, 43, 45, 48, 63, 65), subgroup 3 (“PKDL qPCR LAMP: Blood” consisting of datasets 28, 31 and 40), subgroup 4 (“CL Microscopy LAMP: Skin tissue” consisting of datasets 6, 52, 57, 60, 61, 80) and subgroup 5 (“CL PCR LAMP: Skin Tissue” consisting of datasets 4, 7, 29, 46, 53, 58, 62, 56) were used. For each subgroup of interest, diagnostic test results per patient tested were included more than once only if multiple samples of the same patients were taken at different time points (datasets 30, 31, 46), such as before and after treatment (at follow-up). For several included studies more than one diagnostic test result per patient was available, for example due to multiple LAMP tests with different primer pairs of the same patient sample set. The decision as to which datasets were included was based mainly on the aim of combining similar studies in the subgroups (e.g. same sample type). In addition, arbitrary reasons, such as which datasets best reflected target conditions, determined the choice of datasets. For example, an analysis of a panel of patient samples was conducted in both Suriname (datasets 52 and 53) and the Netherlands (datasets 54 and 55), but only datasets of the endemic country (Suriname) were used for the subgroup analysis. For different primer pairs (datasets 32 and 33) targeting the internal transcribed spacer 1 (*ITS1)* sequence, the dataset with higher sensitivity was included for analysis.

Pooled estimates for S and E of subgroups, *I*^2^ and Tau-squared parameters were calculated using Comprehensive Meta-Analysis version 3.3.070 [93, 94].

Summary receiver operating characteristic (SROC) curves and area under the curve (AUC) of subgroups 1, 2, 4 and 5 were calculated using R using the mada package version 0.5.10 [94] which is based on a bivariate random-effects model [95]. For studies with 2 × 2 tables that contain entries of the value 0, in accordance with the package manual, continuity correction based on Haldane and Ascombe of adding 0.5 to all values of the affected tables was used [96, 97].

## Results

### Literature search

**Fig. 1:**
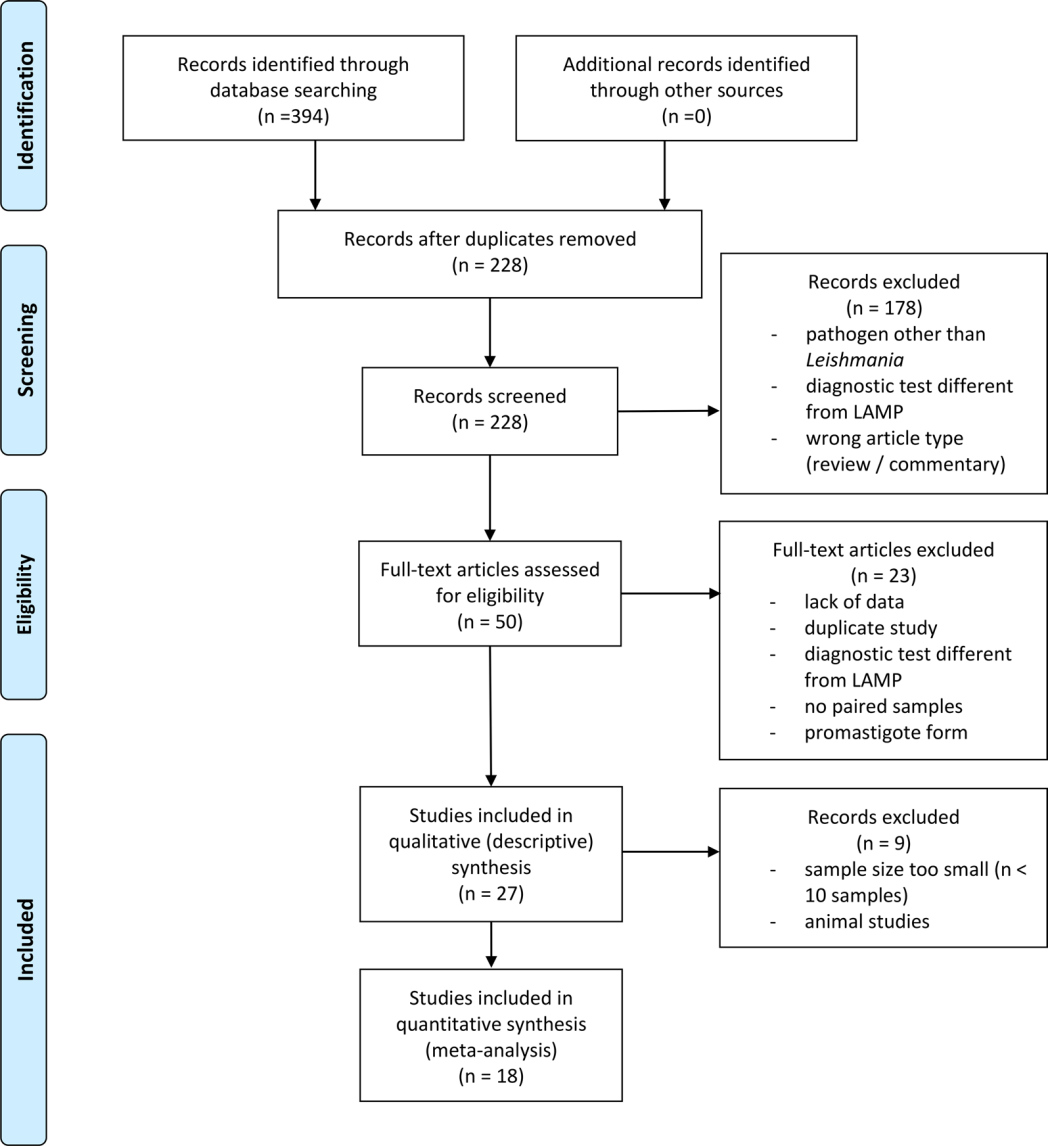
PRISMA flowchart. Literature databases were searched using the defined search strategy, and, after deduplication, the 228 references obtained were screened. Further details on the applied inclusion criteria can be found in the text. Data were extracted for qualitative (descriptive) synthesis (27 studies) and, if eligible, quantitative synthesis (18 studies), which refers to pooled analysis and SROC curves in subgroups

The full workflow of the literature search, based on the principles of the PRISMA guidelines for systematic reviews [83], is shown in Fig. 1.

A total of 394 publications were retrieved; after deduplication, 228 publications were screened by title and abstract and 50 by full text based on the inclusion/exclusion criteria as detailed in the “Methods” section. Studies were excluded at the title/abstract screening stage for the following reasons: wrong pathogen (condition under investigation of the study was not caused by *Leishmania* sp.), no LAMP (LAMP was not used as a diagnostic test method) or wrong article type (reviews and commentaries were excluded, but references were screened for further studies). Studies were excluded at the full-text assessment stage for the following reasons: lack of data (inability to complete a 2 × 2 contingency table), duplicate study (the same clinical data were described in another study), no LAMP (LAMP was not used as a diagnostic test method), no paired samples (samples tested with LAMP and the reference standard were not from the same individuals) or promastigote form (test samples were derived from the promastigote form). Twenty-seven studies were accepted for further analysis and dataset extraction—22 studies regarding leishmaniasis diagnosis in humans [52, 69, 88, 98–116] (Additional file 3: Table S2) and five studies addressing diagnosis in animals (Table 1). For the extracted variables *infecting species* and *readout methods*, we used (*indicated*) if not mentioned directly in the text. For the infecting species, this refers to identification through for example the use of specific primer pairs or epidemiological data without confirmation by further analysis. For the readout method, this refers to identification through specific reagents/kits used.

**Table 1:** Main methodological characteristics of studies addressing leishmaniasis in animals

Author, year	Country	Clinical condition	*Leishmania* species	LAMP target	Animal species	Sample size (cases/controls)	Reference test
Celeste et al. [117]	Laboratory animals	CL	*L. amazon-ensis*, *L. infantum*	kDNA	*Mesocricetus auratus* (hamster)	18/4	PCR PCR-RFLP
Gao et al. [118]	China	VL CL	*L. infantum*	kDNA	*Canis familiaris* (dog)	111/30	Microscopy PCR
Chaouch et al. [119]	Tunisia	VL CL	*L. infantum*	*cpb* gene, 18S rRNA^a^	*Canis familiaris* (dog)	75	Microscopy PCR
Alam et al. [120]	Bangladesh	VL	*L. donovani*	nd	*Bos indicus* (cattle)	11	LnPCR
Maurelli et al. [121]	Italy	VL CL	*L. infantum* (indicated)	18S rRNA	*Canis familiaris* (dogs)	60	qPCR

Study design: consecutive (suspected animals, decision on diseases status is done after recruiting) or case–control (animals were split into a case and a control group)

^a^Additional study data received from authors upon request. VL, visceral leishmaniasis; CL, cutaneous leishmaniasis; PCR, polymerase chain reaction; qPCR, quantitative PCR; LnPCR, nested PCR; PCR-RFLP, polymerase chain reaction-restriction fragment length polymorphism; kDNA, kinetoplast DNA; rRNA, ribosomal RNA; *cpb* gene, cysteine protease B multi-copy gene; nd, no data

### Characteristics of included studies

The datasets were stratified by clinical condition, sample type and reference test used. A full list of datasets per study is available as supplementary information (see Additional file 2: Table S1). Eighty-one and 12 datasets were constructed based on the included studies for LAMP diagnosis in humans and animals, respectively. In the case of missing data for completion of a 2 × 2 contingency table, or a need for clarification, the corresponding and/or first authors of 13 publications were contacted, enabling seven additional datasets to be constructed.

The following descriptions are based on the included human studies, where the following studies are counted more than once as different indications are analysed: Adams et al. [69] two studies (VL and CL), Verma et al. [98] three studies (VL, PKDL and CL), Verma et al. [99] two studies (VL, PKDL), and Sriworarat et al. [100] two studies (VL and CL), resulting in 27 studies in total. In total, 2255 individuals and 6159 test results for diagnosis of leishmaniasis in humans are included in this review. Of the individual tests, 1453 are for diagnosis of VL and 650 of CL. The studies were performed from 2009 to 2019, and about half of them (*n* = 14) during the past 4 years (2017–2020). Out of 27 studies, 21 (78%) evaluated the LAMP performance in the Old World, while four studies evaluated the LAMP performance in New World countries (Brazil, Colombia and Suriname) [52, 69, 101, 102], and one study included a travel case from Venezuela [79]. For two studies the origin of patients is not mentioned. Eighteen studies (67%) used a control group while nine (33%) were categorized as consecutive. Two studies included analysed LAMP performance in PKDL diagnosis. Twenty-three studies (85%) used a commercial kit for DNA extraction; in seven (26%) the kit used was the QIAamp^®^ DNA Blood mini kit (QIAGEN, Hilden, Germany), and six (22%) used a commercial kit for LAMP, which was the Loopamp™ *Leishmania* detection kit (Eiken Chemical, Tokyo, Japan). In 12 cases. *L. donovani* was found or indicated (e.g. through usage of species-specific primer pairs) as the infecting species, *L. tropica* was found in three studies, and *L. infantum*, *L. major* and *L. guyanensis* were found or indicated in two studies each. In 11 studies (40%) the target was kinetoplast DNA (kDNA); in seven (26%) the targets for LAMP were a combination of 18S ribosomal RNA (rRNA) and kDNA genes. The cysteine proteinase b (*cpb*) gene, *ITS1* DNA sequences and *k26* were used in one study each as the targets. In 23 studies (85%) a PCR method (PCR, qPCR or LnPCR) was used as a reference standard, and in 21 (78%) a microscopy method (microscopy or culture microscopy) was used as a reference standard.

The sample size of the 27 included studies ranges from two to 274, with a median of 72 and an interquartile range from 38 (25th percentile) to 95.5 (75th percentile).

### QUADAS-2 based quality assessment

The quality of included studies was analysed based on the QUADAS-2 tool [87]; the results separated by VL and CL diagnosis studies are shown as supplementary information (see Additional file 4: Figure S1). The risk regarding applicability of (1) reference standard, (2) index test and (3) patient selection were judged as low for the included studies. For index and reference test, the risk of bias is unclear in most included studies, with some having a high risk of bias regarding the categories flow and timing and patient selection.

### Performance of LAMP for the diagnosis of leishmaniasis

The forest plots for the S and E of LAMP vs the reference test per dataset are given as supplementary information (see Additional file 5: Figure S2). Spearman’s rank correlation coefficient evaluating the correlation between S and sample size is *r*_s_(S,*n*) = −0.45 (95% CI −0.67 to 0.24) including all studies, compared to *r*_s_(S,*n*) = −0.02 (95% CI −0.31 to 0.29) excluding studies with a sample size ≤ 10, indicating a risk of moderate bias in the case of smaller sample sizes. For E, the correlation coefficient is *r*_s_(E,*n*) = −0.13 (95% CI −0.41 to 0.14) if all studies are included and *r*_s_(E,*n*) = −0.16 (95% CI −0.45 to 0.14) excluding studies with a sample size ≤ 10, indicating a low risk of bias in both cases [122]. For the pooled estimates, we therefore excluded smaller studies with a sample size ≤ 10.

**Fig. 2:**
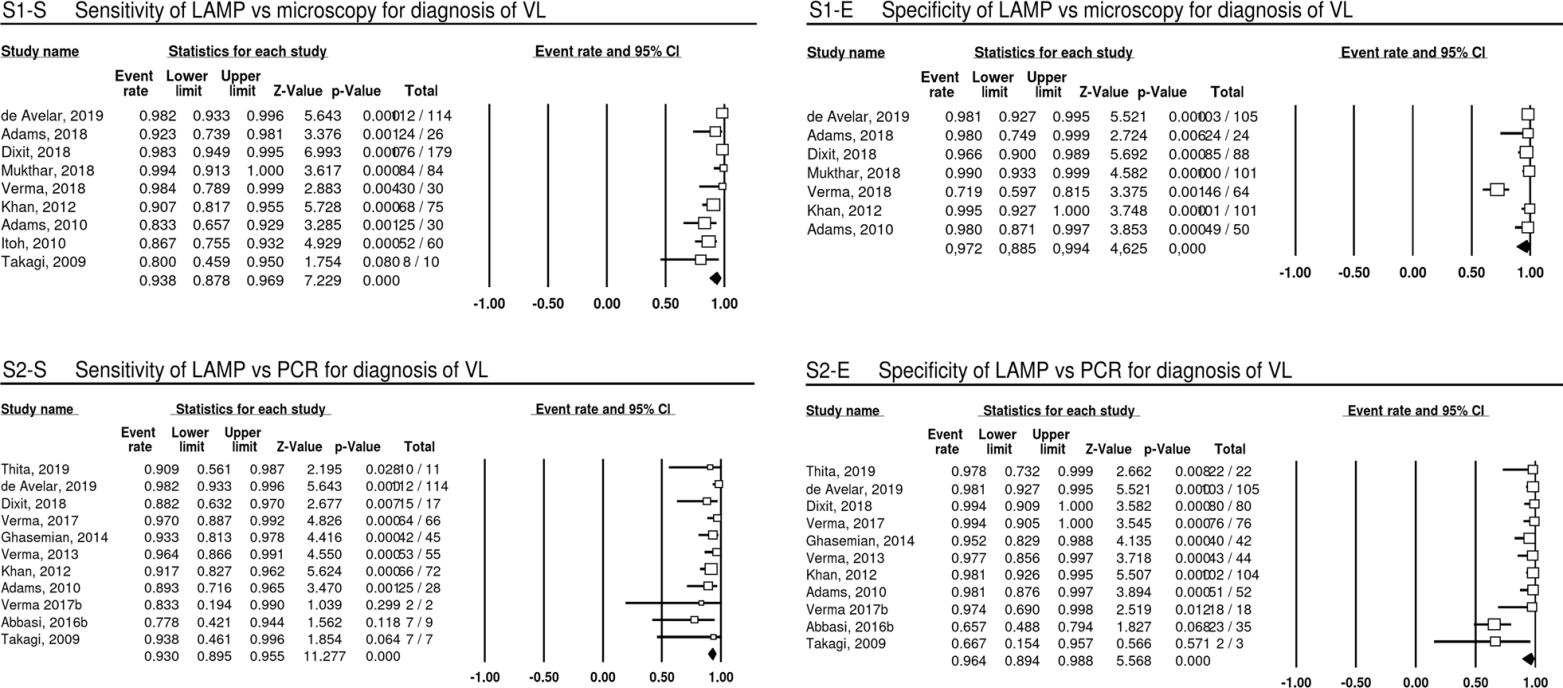
Point and pooled estimates of sensitivity and specificity for studies included in the meta-analysis for diagnosis of VL. Values and pooled estimates (last row per analysis, black diamond) for sensitivity (S1-S) and specificity (S1-E) for subgroup 1 (LAMP compared with microscopy for VL diagnosis) and sensitivity (S2-S) and specificity (S2-E) for subgroup 2 (LAMP compared with PCR methods for VL diagnosis)

Depending on the disease (VL, CL, PKDL), reference standard used (microscopy, PCR methods [PCR, qPCR, LnPCR were grouped together] and qPCR in the case of PKDL) and sample type for LAMP, datasets were combined and are shown under the respective heading. Pooled estimates for S and E of subgroups are shown in Fig. 2a–c. The pooled estimates for S are > 90% for all subgroups except subgroup 4 (LAMP compared with microscopy for CL diagnosis). For VL diagnosis compared to either of the two reference standards (microscopy, PCR) and PKDL diagnosis compared to qPCR, the pooled estimate for E is > 95%. The pooled estimate for subgroup 4 (specificity of LAMP for CL diagnosis compared to microscopy) is 67% (95% CI 45–84%), much lower than any other pooled estimate value.

### LAMP for diagnosis of VL

Compared to microscopy as a reference standard using the sample types bone marrow aspirates (BMA), splenic aspirates (SA) or lymph node aspirates (LNA) for VL diagnosis (subgroup 1) (Fig. 2, S1), datasets (*n* = 9) show S values for LAMP using blood as sample type ranging from 80 to 99% (pooled estimate 93.8%, 95% CI 87.8–96.9%) and E (*n* = 7) from 72 to 100% (pooled estimate 97.2%, 95% CI 88.5–99.4%; two datasets did not contain values for E). Test results for 1141 individual tests are contained in subgroup 1, and the values for *I*^2^ and Tau-squared are 67.78 and 0.76 for the S analysis and 86.55 and 3.22 for the E analysis.

Compared to PCR methods (PCR, qPCR, LnPCR) as reference standards where both tests used blood samples for VL diagnosis (subgroup 2) (Fig. 2, S2), datasets (*n* = 11) show an S ranging from 83 to 98% (pooled estimate 93.0%, 95% CI 89.5–95.5%) and E ranging from 66–99% (pooled estimate 96.4%, 95% CI 89.4–98.8%) for LAMP. Results of 1007 individual tests are contained in subgroup 2, and the values for *I*^2^ and Tau-squared are 9.86 and 0.06 for the S analysis and 79.49 and 2.75 for the E analysis.

### LAMP for diagnosis of PKDL

**Fig. 3:**
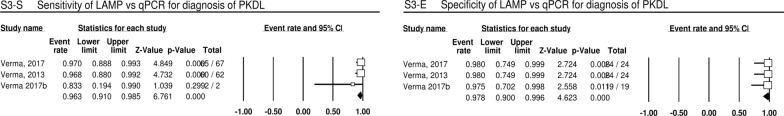
Point and pooled estimates of sensitivity and specificity for studies included in the meta-analysis for diagnosis of PKDL. Values and pooled estimates (last row per analysis, black diamond) for sensitivity (S3-S) and specificity (S3-E) for subgroup 3 (LAMP compared with qPCR for diagnosis of PKDL)

Compared to qPCR as a reference standard where both tests used tissue biopsy samples for PKDL diagnosis (subgroup 3) (Fig. 3, S3), datasets (*n* = 3) show an S ranging from 83–97% (pooled estimate 96.3%, 95% CI 91.0–98.5%) and an E of 98% (pooled estimate 97.8%, 95% CI 90.0–99.6%) for LAMP. Test results of 198 individual tests are contained in subgroup 3, and the values for *I*^2^ and Tau-squared are 0.00 and 0.00 for the S analysis and 0.00 and 0.00 for the E analysis.

### LAMP for diagnosis of CL

**Fig. 4:**
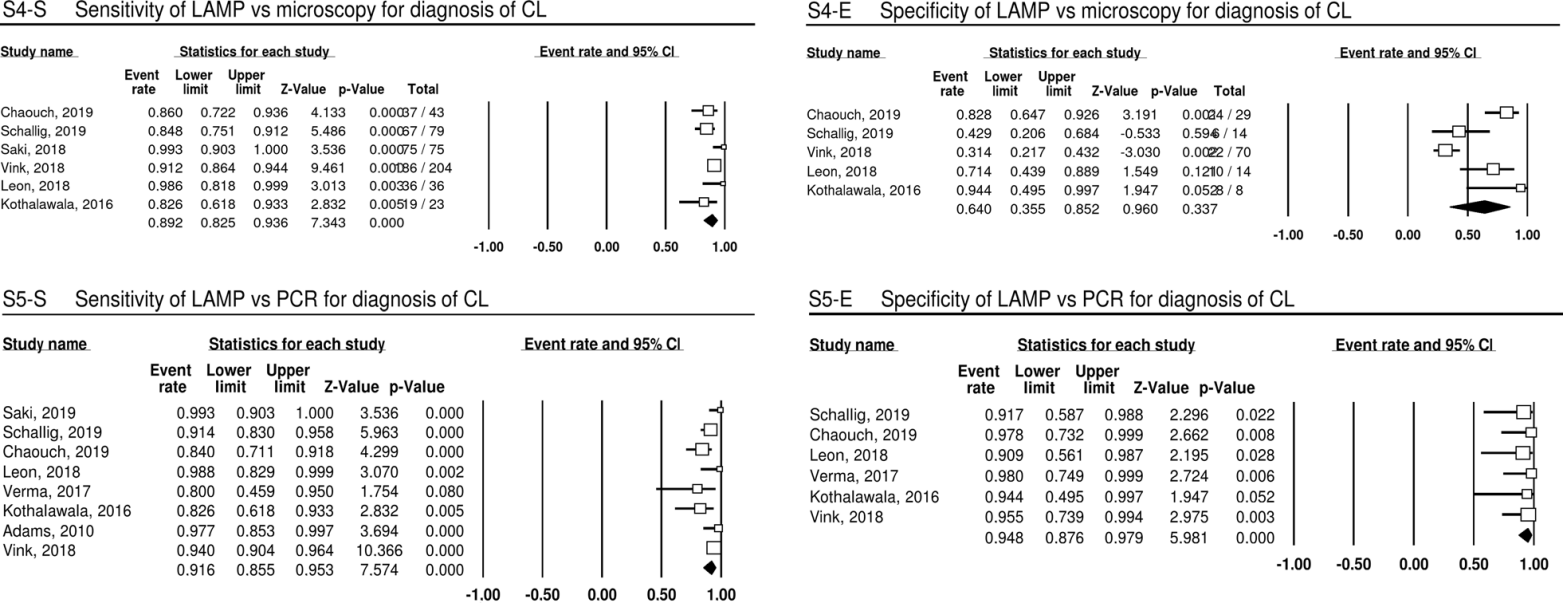
Point and pooled estimates of sensitivity and specificity for studies included in the meta-analysis for diagnosis of CL. Values and pooled estimates (last row per analysis, black diamond) for sensitivity (S4-S) and specificity (S4-E) for subgroup 4 (LAMP compared with microscopy for CL diagnosis) and sensitivity (S5-S) and specificity (S5-E) for subgroup 5 (LAMP compared with PCR methods for CL diagnosis)

Compared to microscopy as reference standard (subgroup 4) (Fig. 4, S4), datasets show an S (*n* = 6) ranging from 83 to 99% (pooled estimate 89.2%, 95% CI 82.5–93.6%) and E (*n* = 5) ranging from 31 to 94% (pooled estimate 64.0%, 95% CI 35.5–85.2%; one dataset did not contain values for E) for LAMP. Test results of 687 individual tests are contained in subgroup 4, and the values for *I*^2^ and Tau-squared are 51.63 and 0.22 for the S analysis and 84.39 and 1.37 for the E analysis.

Compared to PCR variations (PCR, qPCR, nested PCR) as a reference standard (subgroup 5) (Fig. 4, S5), datasets (*n* = 8) show an S ranging from 80–99% (pooled estimate 91.6%, 95% CI 85.5–95.3%) and E ranging from 91–98% (pooled estimate 94.8%, 95% CI 87.6–97.9%) for LAMP. Test results of 672 individual tests are contained in subgroup 5, and the values for *I*^2^ and Tau-squared are 57.73 and 0.38 for the S analysis and 0.00 and 0.00 for the E analysis.

### LAMP for diagnosis of CL and VL in animals

In general, few studies reported data on leishmaniasis in animals.

Compared to microscopy as a reference standard, datasets (*n = 3*, III, V and VIII) show an S ranging from 54 to 100% and an E ranging from 43%–77% for LAMP.

Compared to PCR variations (qPCR, PCR-RFLP) as a reference standard, datasets (*n* = 9, numbers I, II, IV, VI, VII and–IX-XII) show an S ranging from 0 to 100% and an E ranging from 50 to 100% for LAMP.

In line with human studies, if datasets are derived from the same individuals within the same study, only those datasets with the reported higher S were considered.

The three datasets comparing LAMP to microscopy are part of two separate studies investigating canine leishmaniasis, CL and VL, in 186 animals [118, 119]. Datasets III and VIII report an S of 100% (95% CI 74–100%) and 68% (95% CI 49–83%) and an E of 43% (95% CI 33–54%) and 77% (95% CI 61–89%), respectively.

Six datasets (IV, VI, VII, X-XII), part of three studies [118, 119, 121], compare LAMP to PCR for investigation of canine leishmaniasis (CL and VL) in a total of 279 animals. Datasets IV, VI and XII report an S of 100% (95% CI 95–100%), 75% (95% CI 51–91%) and 91% (95% CI 59–100%), and an E of 91% (95% CI 77–98%), 78% (95% CI 65–88%) and 96% (95% CI 86–100%), respectively. One study (dataset IX, [120]) investigated VL in domestic cattle and only reported negative cases. Two datasets (I and II), part of one study [117], reported data from CL in Syrian hamsters, with a reported S of 89% (95% CI 65–99%) and an E of 100% (95% CI 40–100%) for dataset I and an S of and 100% (95% CI 59–100%) and an E of 50% (95% CI 1–99%) for dataset II, which only analysed seven samples.

Due to the great heterogeneity with regard to animal species, forms of leishmaniasis (CL vs VL) and sample types, no pooled analysis was conducted.

### Analysis of LAMP performance using SROC curves

**Fig. 5:**
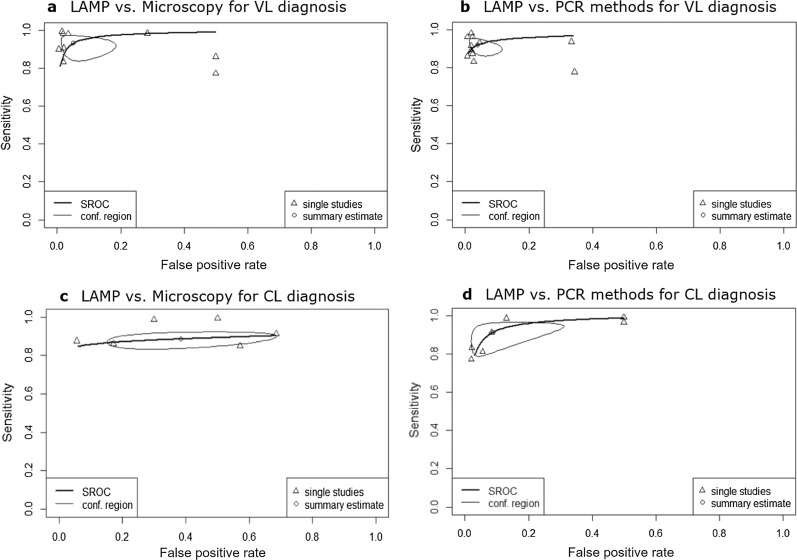
SROC curves. Comparison of LAMP with microscopy (**a**) and PCR (**b**) for VL diagnosis, and microscopy (**c**) and PCR (**d**) for CL diagnosis, using SROC curves. Arrows represent the single study data, and circles indicate summary estimates with 95% confidence regions

Based on the subgroups, where similar studies such as LAMP used blood samples for diagnosis of VL compared to microscopy, analyses using SROC curves were performed. The SROC curves for different sample types comparing LAMP with microscopy and PCR are shown in Fig. 5. The AUC values are 0.973 (subgroup 1), 0.960 (subgroup 2), 0.881 (subgroup 4) and 0.964 (subgroup 5), indicating that LAMP is a highly sensitive and specific diagnostic test for VL, PKDL and CL.

## Discussion

Leishmaniasis is considered a neglected tropical disease with various clinical manifestations endemic in more than 80 countries. Early diagnosis and treatment is not only of utmost importance for the individual but also for the community as key components of leishmaniasis control [123]. Since its invention, LAMP, a modification of the PCR protocol, has been described as a very robust and specific molecular diagnostic method due to the primer and amplification structure used [56]. General advantages further include easy readout methods through visibility of reaction by-products such as turbidity [65], or addition of different dyes [69, 101, 103].

In this section, we will discuss characteristics of the included studies and the performance of LAMP for the diagnosis of VL and CL, as well as the observed heterogeneity among the datasets. This is followed by an assessment of the implementability of LAMP in the diagnostic workflow, a brief discussion of the importance of diagnosis of leishmaniasis in animal hosts, and concluded by the study’s strengths and limitations.

The pooled estimates of the subgroups comparing LAMP with microscopy/PCR for VL/CL diagnosis were > 90% for sensitivity and > 95% for specificity, except for LAMP compared to microscopy for CL diagnosis (subgroup 4), where specificity was found to be 64%, therefore only moderate. These results correspond to the calculated AUC values which are > 0.96, except for the same subgroup 4, where an AUC value of 0.881 was found. This subgroup 4 consisted of six studies for a total of 687 individual tests performed, giving a broad 95% confidence interval from 35 to 85% for specificity. This result deserves reflection. Considering the known low sensitivity of the direct microscopic test, this low specificity may demonstrate not a failure but a superior performance of the LAMP, capable of identifying true cases which are erroneously counted as false positives due to the reference test being microscopy.

To overcome this issue, a composite reference standard could be used, such as that by Vink et al. [88]. In this study, considerably more positive cases were detected by the molecular method than by microscopy (out of the 257 considered true cases, 252 were positive by qPCR and 204 by microscopy). Alternatively, statistical methods such as latent class modelling have been used in the absence of a gold standard for diagnosis [124, 125].

We found that for most subgroups the observed heterogeneity can be attributed to differences between the studies rather than sampling error only [126]. The calculated *I*^2^ values were > 0.1 for most subgroup analyses, except for subgroup 3 (LAMP compared with qPCR for PKDL diagnosis) and subgroup 2 (LAMP compared with PCR for VL diagnosis) with regard to sensitivity, and subgroup 5 (LAMP compared with PCR for CL diagnosis) with regard to specificity. Heterogeneity in the subgroups may be due to several factors potentially influencing the results of an analytical method. We found little data dedicated to the study of robustness of LAMP in the context of leishmaniasis diagnosis [101, 104, 127], and some parameters, such as stability of DNA contained in clinical samples, inter-operator reliability or operator training (e.g. new method vs a method well established in the conducting laboratory), were rarely reported in studies. Further validation studies using standardized protocols and conducted in endemic countries would enable better comparisons and support decision-making in relation to diagnostic algorithms in different scenarios. We further recommend including individual sample data for publication, in order to allow statistical meta-analyses.

Parameters possibly influencing LAMP performance are sample type, DNA extraction method, target sequence and readout method (see Table 1).

Molecular targets, and the variety of suitable markers, for *Leishmania* species have been discussed in detail in Akhoundi et al. [36]. The most frequently used targets in the studies included were kDNA and 18S rRNA, the structural RNA of the ribosomal small subunit. 18S rRNA has the advantage of being a candidate for pan-*Leishmania* assays due to sections of high sequence conservation between species [100, 128]. To a lesser extent, *ITS1*, *cpb*, *k26* and *L151* were also used. In general, primers must be designed carefully and, if possible, tested in silico and in vitro, as cross-reactivity with other closely related genera such as *Trypanosoma* has been observed in some studies [69, 100, 129]. The impact of this cross-reactivity could be reduced by taking into account different clinical presentations of patients [69, 100]. Special consideration should be applied to endemic areas of South America, where co-infections of leishmaniasis and Chagas disease infections caused by *Trypanosoma cruzi* are possible, as endemic areas of the respective pathogens overlap [130].

An overview and evaluation of different readout methods can be found in Nzelu et al. [131]. LAMP results can be interpreted visually by turbidity or colour change, which is used in the majority of studies. In some studies, positive samples are confirmed by gel electrophoresis [101, 105–108]. However, opening of tubes after the reaction bears the risk of introducing amplicon contamination and should therefore be conducted only with caution and suitable internal quality controls [58–60].

In most studies included (85%), commercial kits were used for DNA extraction, which offer the advantage of better reproducibility, but could be less suitable for a PoC setting due to equipment requirements. Some studies also used a “direct boil-and-spin” approach [100, 103, 109]: whole blood was centrifuged after addition of a lysis agent and heating. The results were found to be comparable to other LAMP protocols involving more sophisticated DNA extraction and purification (Figs. 2, 3, 4), and are also in line with studies such as Nzelu et al. [128], but further studies using clinical samples would be needed for confirmation. Depending on the desired level of implementation, an evaluation of a “LAMP near-PoC” method focusing on using as little equipment as possible, for example the usage of electricity-free heat sources (such as the non-instrumented nucleic acid amplification [NINA] device [132] or commercial pocket warmers [133]), might provide valuable insights. Protocols without kits and low laboratory equipment requirements favour the cost–benefit ratio compared to other molecular methods, making LAMP a cost-effective diagnostic method [134].

The desired parameters of a diagnostic test strongly depend on the intended usage [135]. As molecular diagnostic tests can have very high analytical sensitivity, they correlate better with infection status than actual disease [6]. There are several possible reasons that the identification of asymptomatic individuals might also be desired. First of all, epidemiological prevalence studies allow for effective regional disease monitoring, and might support related decisions, for example the identification of areas where prophylactic measures (such as the usage of bed nets or insecticide-impregnated fly screens) should be promoted [136]. Furthermore, in the context of blood donations, a method with high analytical sensitivity is desired. Contaminated blood products pose a potential risk of transmission, particularly for immunocompromised blood recipients [137]. Related to epidemiological prevalence studies in humans, another possible area of applicability includes xenomonitoring, where a large quantity of samples can be analysed in a short time using a pooling approach [128, 131].

A guideline to aid in selecting the optimal diagnostic test for an intended purpose was published by WHO, reporting the ASSURED criteria (Affordable, Sensitive, Specific, User-friendly, Rapid and robust, Equipment-free and Deliverable) and their adaptation to fit each diagnostic need, also taking into account special requirements for PoC diagnostic tests [138–141]. This guideline suggests six evaluation steps, starting with defining the test purpose, comparing characteristics of available products, reviewing the regulatory approval, obtaining data under first, ideal, and second, real conditions and finally, monitoring the test performance in routine use.

Unfortunately, we were only able to report a limited number of studies using LAMP for the diagnosis of CL and VL in animals, and due to the heterogeneity in terms of species, forms of leishmaniasis and sample types, no pooled analysis was conducted.

This is particularly disappointing, since the failure of leishmaniasis control is partially associated with a failure of control of infected animal hosts, such as dogs in domestic settings [142, 143]. Taking Brazil as an example, high costs for control and prevention of canine leishmaniasis have been reported previously, which are in contrast to the limited financial resources for control programmes in endemic areas [144–146]. In addition, current available serological screening tests for canine leishmaniasis present a certain level of disagreement [147]. Therefore, research into highly sensitive and specific as well as affordable methods for diagnosis of leishmaniasis in animal hosts, most importantly dogs, is very much needed and crucial for control efforts.

In summary, our results show LAMP to be a suitable candidate for a PoC-test in human patients, but further research and matching against actual requirements is needed. For example, we found LAMP to only partly cover the requirements for a PoC test for CL, such as minimum sensitivity of 85% and minimum specificity of 90%, and other parameters covered in a comprehensive target product profile developed by the Foundation for Innovative New Diagnostics (FIND) [148].

In our opinion, the strengths of this literature review and meta-analysis are the comprehensive search strategy and the number of databases included in the literature search. In addition, we aimed to include unpublished data (e.g. conference abstracts) and contacted authors; thus, a number of additional datasets could be collected.

The most important limitation of this literature review and meta-analysis is the heterogeneity for most analyses based on our results; consequently, the results have to be interpreted with caution [149]. In addition, the risk of bias was evaluated, and many of the included studies have unclear and/or high risk of bias for the evaluated parameters of “patient selection” and “flow and timing”. Moreover, although we aimed to exclude patient samples that were used in several studies, we were unable to do so and therefore decided to include a subset of VL and PKDL samples that were analysed in two studies by Verma et al. [98, 99].

## Conclusions

In summary, LAMP has high sensitivity and specificity compared to microscopy and PCR methods for diagnosis of CL, PKDL and VL. An advantage of LAMP which is shared by other molecular methods is the possibility to use minimally and non-invasive sample types, such as whole blood for VL and swabs for CL diagnosis. Advantages more specific to LAMP are the high robustness and isothermal amplification, so LAMP could be conducted with unpurified or minimally purified samples and with heat sources not relying on electricity, which could be interesting in a (near-)PoC setting. Currently, LAMP seems to be a suitable diagnostic test in prevalence studies, epidemiological studies (in humans and animals) and diagnosis in a diagnostic algorithm, especially for immunocompromised patients, or possibly for monitoring therapeutic success. Our findings are limited by the rather low number of studies available; thus, further large-scale studies evaluating LAMP in field settings, complemented by cost-effectiveness analyses, are recommended to gain further insights.

## Supplementary Information

**Additional file 1: Text S1.** Search strategy and results per database. Shows the specific search strategy used for the databases included in the review, the search dates and the number of results per database.

**Additional file 2: Table S1.** Datasets of included studies addressing the diagnosis of leishmaniasis in humans and animals.

**Additional file 3: Table S2.** Main methodological characteristics of studies addressing the diagnosis of leishmaniasis in humans.

**Additional file 4: Figure S1.** QUADAS-2 based quality risk assessment.

**Additional file 5: Figure S2.** Forest plots for sensitivity and specificity for all identified datasets.

## Data Availability

The datasets supporting the conclusions of this article are included within the article and its additional files.

## Abbreviations

ASSURED: Affordable, Sensitive, Specific, User-friendly, Rapid and robust, Equipment-free and Deliverable
AUC: Area under the curve
BMA: Bone marrow aspirates
CI: Confidence interval
CL: Cutaneous leishmaniasis
CSFV: Classical swine fever virus
*cpb*: Cysteine proteinase b
DAT: Direct agglutination test
DNA: Deoxyribonucleic acid
DND*i*: Drugs for Neglected Diseases *initiative*
dsDNA: Double-stranded DNA
DOR: Diagnostic odds ratio
E: Specificity
FN: False negative
FP: False positive
FIND: Foundation for Innovative New Diagnostics
INF: Infinite
*ITS1*: Internal transcribed spacer 1
kDNA: Kinetoplast DNA
LAMP: Loop-mediated isothermal amplification
LNA: Lymph node aspirates
LnPCR: Nested PCR
LR−: Negative likelihood ratio
LR+: Positive likelihood ratio
MCL: Mucocutaneous leishmaniasis
n.d./n.D.: No data
NaN: Not a number
NINA: Non-instrumented nucleic acid amplification
NPV: Negative predictive value
PBMC: Peripheral blood mononuclear cells
PCR: Polymerase chain reaction
PCR-RFLP: PCR restriction fragment length polymorphism
PRISMA: Preferred reporting items for systematic reviews and meta-analyses
PKDL: Post-kala-azar dermal leishmaniasis
PoC: Point of care
PPV: Positive predictive value
qPCR: Quantitative real-time polymerase chain reaction
qRT-PCR: Quantitative reverse transcriptase PCR
RDT: Rapid diagnostic test
RNA: Ribonucleic acid
RT: Reverse transcriptase
rRNA: Ribosomal RNA
RS: Reference standard
SAM: Splenic aspiration microscopy
S: Sensitivity
SA: Splenic aspirates
SROC: Summary receiver operating characteristic
TN: True negative
TP: True positive
VL: Visceral leishmaniasis
WHO: World Health Organization
95 L: 95% Confidence interval lower limit
95 H: 95% Confidence interval higher limit
