# Supplementary material for: Diagnosis of visceral and cutaneous leishmaniasis using loop-mediated isothermal amplification (LAMP) protocols: a systematic review and meta-analysis
Source: Parasit Vectors. 2022 Jan 24;15:34. doi: 10.1186/s13071-021-05133-2 (PMC8785018; doi:10.1186/s13071-021-05133-2)
Supplement: Supplementary file 1 — Additional file 1: Text S1. Search strategy and results per database. Shows the specific search strategy used for the databases included in the review, the search dates and the number of results per database. [file 13071_2021_5133_MOESM1_ESM.pdf]

Additional file 1: Text S1. Search strategy and results per database

Search strategy per database

1. Web of Science Core Collection

(„Leishmania*“ AND („Loop*mediated” OR (“Isothermal” AND “Amplification”)))

All languages & All document types

All years (1899-2019)

2. Epistemonikos

(title:((title:(Leishmania*) OR abstract:(Leishmania*)) AND (title:(amplification) OR abstract:(amplification))) OR abstract:((title:(Leishmania*) OR abstract:(Leishmania*)) AND (title:(amplification) OR abstract:(amplification))))

Languages: English

Title/Abstract

Publication year: All

Added to database: All

Publication type: All

Pubmed central (PMC): All

3. Global Index Medicus

(tw:(Leishmania*)) AND (tw:((Loop*mediated OR (Isothermal AND Amplification))))

Title, abstract, subject

4. PubMed

(Leishmania*) AND (Loop*mediated OR (Isothermal AND Amplification))

(leishmania[All Fields] OR leishmania'[All Fields] OR leishmania's[All Fields] OR leishmania,[All Fields] OR leishmaniaafter[All Fields] OR leishmaniaamazonensis[All

Fields] OR leishmaniaand[All Fields] OR leishmaniaantigen[All Fields] OR leishmaniabraziliensis[All Fields] OR leishmaniac[All Fields] OR leishmaniac'[All Fields] OR leishmaniachagasi[All Fields] OR leishmaniacidal[All Fields] OR leishmaniacides[All Fields] OR leishmaniacs[All Fields] OR leishmaniacs'[All Fields] OR leishmaniadeoxyribonucleic[All Fields] OR leishmaniadonovani[All Fields] OR leishmaniae[All Fields] OR leishmaniaenriettii[All Fields] OR leishmaniaguyanensis[All Fields] OR leishmaniain[All Fields] OR leishmaniainfantum[All Fields] OR leishmaniais[All Fields] OR leishmaniaisis[All Fields] OR leishmanial[All Fields] OR leishmanials[All Fields] OR leishmaniamajor[All Fields] OR leishmaniamexicana[All Fields] OR leishmaniami[All Fields] OR leishmanian[All Fields] OR leishmaniao[All Fields] OR leishmaniapanamensis[All Fields] OR leishmaniaparasites[All Fields] OR leishmanias[All Fields] OR leishmaniase[All Fields] OR leishmaniasen[All Fields] OR leishmaniases[All Fields] OR leishmaniases'[All Fields] OR leishmaniashis[All Fields] OR leishmaniasia[All Fields] OR leishmaniasiamensis[All Fields] OR leishmaniasias[All Fields] OR leishmaniasic[All Fields] OR leishmaniasica[All Fields] OR leishmaniasicas[All Fields] OR leishmaniasis[All Fields] OR leishmaniasis'[All Fields] OR leishmaniasis'i[All Fields] OR leishmaniasis's[All Fields] OR leishmaniasis,[All Fields] OR leishmaniasisde[All Fields] OR leishmaniasise[All Fields] OR leishmaniasisin[All Fields] OR leishmaniasisis[All Fields] OR leishmaniasisleishmania[All Fields] OR leishmaniasisli[All Fields] OR leishmaniasisone[All Fields] OR leishmaniasp[All Fields] OR leishmaniaspp[All Fields] OR leishmaniastrains[All Fields] OR leishmaniasy[All Fields] OR leishmaniasys[All Fields] OR leishmaniatae[All Fields] OR leishmaniatropica[All Fields] OR leishmaniaviannia[All Fields] OR leishmaniavirus[All Fields] OR leishmaniaviruses[All Fields] OR leishmaniazy[All Fields]) AND (Loop*mediated[All Fields] OR (Isothermal[All Fields] AND Amplification[All Fields]))

5. PMC

(Leishmania*) AND (Loop*mediated OR (Isothermal AND amplification))

(leishmania[Abstract] OR leishmania/crithidia[Abstract] OR leishmania/hiv[Abstract] OR leishmania/m[Abstract] OR leishmania/macrophage[Abstract] OR leishmania's[Abstract] OR leishmania5[Abstract] OR leishmaniaand[Abstract] OR leishmaniabraziliensis[Abstract] OR leishmaniacidal[Abstract] OR leishmaniacides[Abstract] OR leishmaniacs[Abstract] OR leishmaniae[Abstract] OR leishmaniagenus[Abstract] OR leishmaniain[Abstract] OR leishmaniainfantum[Abstract] OR leishmaniais[Abstract] OR leishmaniaisis[Abstract] OR leishmanial[Abstract] OR leishmanials[Abstract] OR leishmanian[Abstract] OR leishmaniaprotozoan[Abstract] OR leishmanias[Abstract] OR leishmaniases[Abstract] OR leishmaniasic[Abstract] OR leishmaniasis[Abstract] OR leishmaniasis/hiv[Abstract] OR leishmaniasis'[Abstract] OR leishmaniasisin[Abstract] OR leishmaniasp[Abstract] OR leishmaniaspecies[Abstract] OR leishmaniaspp[Abstract] OR leishmaniavirus[Abstract] OR leishmaniaviruses[Abstract] OR leishmaniawas[Abstract]) AND (Loop*mediated[All Fields] OR (isothermal[All Fields] AND amplification[All Fields]))

6. The Cochrane Library

Leishmania* AND amplification

All Text

7. EMBASE

(Leishmania*) AND (Loop*mediated OR (Isothermal AND Amplification))

8. Scopus

(Leishmania*) AND (Loop*mediated OR (Isothermal AND Amplification))

<u>Overview of search results per database</u>

Initial search date: 7/2019, search updated: 7/2020

Database	Search Results in total
Web of Science Core Collection	72
Epistemonikos	13
Global Index Medicus	3
PubMed	46
PMC	99
The Cochrane Library	3
EMBASE	86
Scopus	72
