# Supplementary material for: Diagnosis of visceral and cutaneous leishmaniasis using loop-mediated isothermal amplification (LAMP) protocols: a systematic review and meta-analysis
Source: Parasit Vectors. 2022 Jan 24;15:34. doi: 10.1186/s13071-021-05133-2 (PMC8785018; doi:10.1186/s13071-021-05133-2)
Supplement: Supplementary file 3 — Additional file 3: Table S2. Main methodological characteristics of studies addressing the diagnosis of leishmaniasis in humans. [file 13071_2021_5133_MOESM3_ESM.pdf]

Additional File 3: Table S2. Main methodological characteristics of studies addressing leishmaniasis in humans.

Data was extracted from studies and included in the qualitative and quantitative synthesis. Study design: Cons (consecutive: suspected individuals, decision on diseases status is done after recruiting) or c-c (case-control: individuals were recruited into a case and a control group). For an explanation of 'indicated', see main text.

**Author, year**	**Country**	**Clinical condition**	**Leishmania species**	**Study design**	**LAMP target**	**Specimen tested**	**Sample size (cases/ controls)**	**Readout method**	**Reference test**
Adams et al., 2018[144]	Ethiopia (VL)	VL	*L.donovani* (indicated)	Cons	18S rRNA kDNA	Whole blood (whole blood, buffy coat, peripheral blood mono-nuclear cells)	50	Visual inspection (color change), UV detection (turbidimeter)	Microscopy
Chaouch et al., 2019* [122]	Tunisia	CL	*L. major* *L. tropica*	Cons	*cpb* gene	Skin tissue (lesion aspirates)	72	Visual inspection (precipitate, indicated) gel electrophoresis	Microscopy qPCR

Leon et al., 2018[101]	Colombia	CL	n/d	Cons	18S rRNA	Tissue samples (Lysed tissue from microscopy slides)	50	Visual inspection (color change), gel electrophoresis	Microscopy qPCR
Dixit et al., 2018[111]	India	VL	*L.donovani*	C-c	kDNA	Whole blood (whole blood)	179 / 88	Visual inspection (color change)	rK39 RDT and/or microscopy qPCR
Ibarra-Meneses et al., 2018[116]	n/d	VL	*L. infantum* (indicated)	C-c	18S rRNA kDNA	Whole blood (whole blood)<br>Tissue samples (BMA)	3 / 2 (per sample type)	UV detection (incubator, fluorimeter)	Microscopy LnPCR qPCR
Mukthar et al., 2018[125]	Sudan	VL	*L. donovani* (indicated)	Cons	18S rRNA kDNA	Whole blood (whole blood, buffy coat)	185	UV detection (incubator)	Microscopy
Verma et al., 2017[98]	India	VL<br>PKDL<br>CL	*L. donovani* (VL, PKDL, indicated)<br>n/d (CL)	C-c	kDNA	Whole blood (whole blood, VL, controls)<br>Tissue samples (BMA (VL))	66 / 76 (VL)<br>15 (bma)<br>67 (PKDL)	Visual inspection (color change)	qPCR, microscopy (VL, PKDL)<br>qPCR (CL)

						Skin tissue (punch biopsy (CL, PKDL, controls))	10 (CL)<br>24 (biopsy, controls)		
Abbasi et al., 2016[121]	Ethiopia	VL	n/d	Cons	*ITS1*	Whole blood (finger prick blood)	44	UV detection, visual inspection (color change), gel electrophoresis	qPCR
Ghasemian et al., 2014[123]	Iran	VL	*L. infantum*	C-c	kDNA	Whole blood (whole blood)	47 / 40	Visual inspection (precipitate, color change), gel electrophoresis	Microscopy<br>LnPCR
Verma et al., 2013[99]	India	VL<br>PKDL	*L. donovani*	C-c	kDNA	Tissue sample (BMA) (VL)<br>Whole blood (whole blood (VL controls))<br>Tissue sample (punch biopsy (PKDL, controls))	15 (BMA)<br>55 /44 (blood)	Visual inspection (color change)	Microscopy and/or PCR

							62 / 24 (tissue biopsy)		
Khan et al., 2012[145]	Bangladesh	VL	*L. donovani*	C-c	kDNA	Whole blood (buffy coat)	75 /101	Visual inspection (precipitate)	Microscopy LnPCR
Adams et al., 2010[69]	Sudan (VL) Suriname (CL)	VL CL	*L. donovani* (VL) *L. guyanensis* (CL)	C-c	18S rRNA	Whole blood (whole blood (VL, controls)) Skin tissue (biopsy (CL))	30 / 50 (VL) 43 (CL)	UV detection (UV lamp)	Microscopy (VL) qPCR (CL)
Takagi et al., 2009[124]	Bangladesh	VL	*L. donovani*	C-c	kDNA	Whole blood (whole blood)	10	Visual inspection (precipitate)	Microscopy PCR LnPCR
Vink et al.*, 2018[88]	Afghanistan	CL	*L. tropica*	Cons	18S rRNA, kDNA	Skin samples (dental broach)	274	UV detection (incubator)	Microscopy and/or PCR
Schallig at al., 2019* [52]	Suriname	CL	*L. guyanensis*	Cons	18S rRNA, kDNA	Skin samples (dental broach)	93	UV detection (turbidimeter)	Microscopy and/or PCR*

			L. amazonensis						
Kothalawala et al., 2016[146]	Sri Lanka	CL	*L. donovani*	Cons	kDNA	Skin samples (aspirates)	31	Visual inspection (precipitate)	Microscopy LnPCR
Itoh and Takagi, 2011[147]	n/d	VL	*L. donovani* (indicated)	C-c	kDNA	Whole blood (whole blood)	60	Visual inspection (precipitate)	Microscopy
Saki et al., 2019[148]	Iran	CL	*L.major*	C-c	18S rRNA	Skin samples (aspirates)	75	Visual inspection (precipitate), gel electrophoresis	Microscopy LnPCR
Thita et al., 2019[149]	Thailand	VL	n/d	C-c	18S rRNA	Whole blood (buffy coat)	33	Visual inspection (color change), UV detection (trans-illuminator), gel electrophoresis	PCR
de Avelar et al., 2019[102]	Brazil	VL	*L. infantum* (indicated)	C-c	*K26*	Whole blood (whole blood)	114 / 105	Visual inspection (precipitate), gel electrophoresis	Microscopy PCR

Sriworarat et al., 2015[100]	Thailand	VL<br>CL	*L. martiniquensis*	C-c	18S rRNA	Whole blood (whole blood), Saliva (VL, CL)<br>Tissue samples (BMA (VL))<br>Tissue sample (biopsy (CL))	2 (VL)<br>1 (CL)<br>1 (control)	Visual inspection (color change), gel electrophoresis	Microscopy<br>qPCR
Imai et al., 2017[103]	CL	Syria<br>Japan / Venezuela	*L. tropica*<br>*L. braziliensis*	C-c	18S rRNA	Swab	2	Visual inspection (color change)	Microscopy<br>PCR

*: Additional study data received from authors upon request

VL (visceral leishmaniasis), CL (cutaneous leishmaniasis), PKDL (Post-kala-azar dermal leishmaniasis), PCR (polymerase chain reaction), qPCR (quantitative PCR), LnPCR (nested PCR), BMA (bone marrow aspirates), LNA (lymph node aspirates), LAMP (Loop-mediated isothermal amplification), kDNA (kinetoplast DNA), rRNA (ribosomal RNA), *cpb* gene (cysteine protease B multi copy gene), *ITS1* (internal transcribed spacer), rK39 RDT (recombinant antigen 39 rapid diagnostic test), n/d (no data).
