# Supplementary material for: Diagnosis of visceral and cutaneous leishmaniasis using loop-mediated isothermal amplification (LAMP) protocols: a systematic review and meta-analysis
Source: Parasit Vectors. 2022 Jan 24;15:34. doi: 10.1186/s13071-021-05133-2 (PMC8785018; doi:10.1186/s13071-021-05133-2)
Supplement: Supplementary file 4 — Additional file 4: Figure S1. QUADAS-2 based quality risk assessment. [file 13071_2021_5133_MOESM4_ESM.pdf]

Additional File 4: Figure S1. QUADAS-2 based quality risk assessment.

Risk of bias was assessed based on the QUADAS-2 tool where the risk of bias regarding different study characteristics (patient selection, index test, reference standard and flow and timing) was evaluated for a) studies regarding VL diagnosis in humans, b) studies for CL diagnosis in humans, c) animal studies. The risk of applicability was considered low for all included studies. VL (visceral leishmaniasis), CL (cutaneous leishmaniasis), RS (reference standard), PCR (polymerase chain reaction)

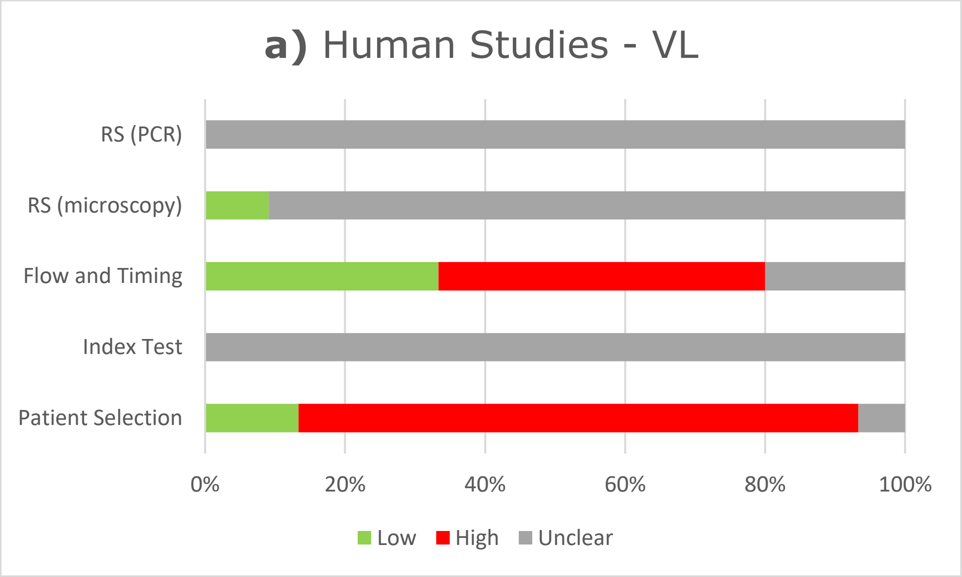

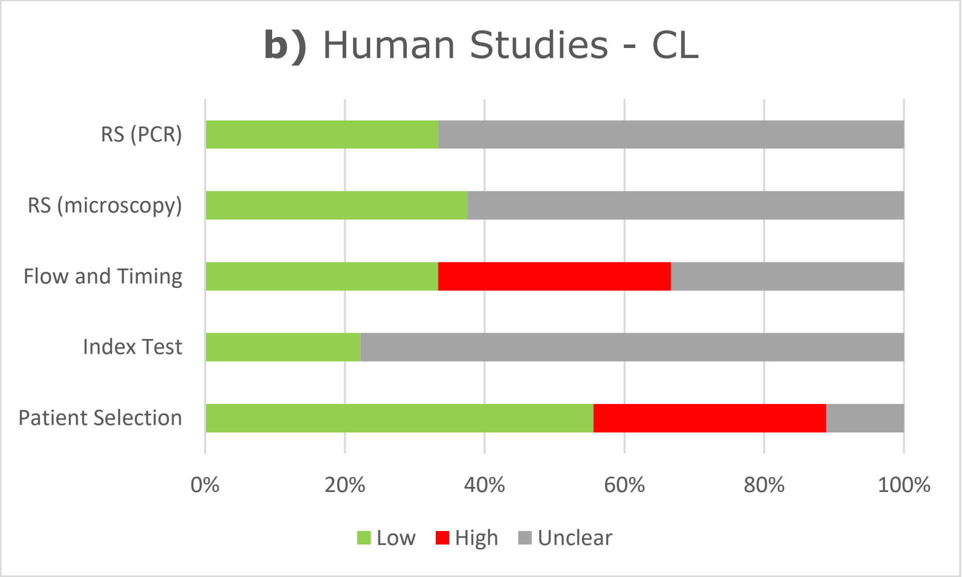

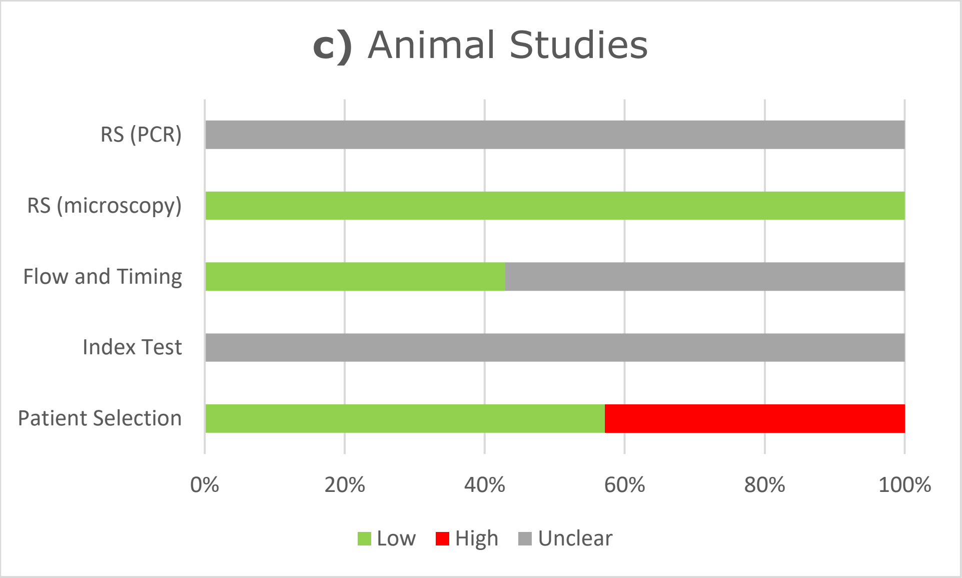
c) Animal Studies
RS (PCR)
RS (microscopy)
Flow and Timing
Index Test
Patient Selection
0%
20%
40%
60%
80%
100%
Low
High
Unclear
