# Supplementary material for: Diagnosis of visceral and cutaneous leishmaniasis using loop-mediated isothermal amplification (LAMP) protocols: a systematic review and meta-analysis
Source: Parasit Vectors. 2022 Jan 24;15:34. doi: 10.1186/s13071-021-05133-2 (PMC8785018; doi:10.1186/s13071-021-05133-2)

Additional File 5: Figure S2. Forest plots of all datasets

LAMP vs. Microscopy for VL diagnosis using whole blood

Study	TP	FP	FN	TN	Sensitivity (95% CI)	Specificity (95% CI)
Adams et al. 2010, Dataset 44	25	1	5	49	0.83 [0.65, 0.94]	0.98 [0.89, 1.00]
Adams et al. 2018, Dataset 1	24	0	2	24	0.92 [0.75, 0.99]	1.00 [0.86, 1.00]
Adams et al. 2018, Dataset 2	23	1	3	23	0.88 [0.70, 0.98]	0.96 [0.79, 1.00]
Adams et al. 2018, Dataset 3	24	1	2	23	0.92 [0.75, 0.99]	0.96 [0.79, 1.00]
de Avelar et al. 2019, Dataset 64	112	2	2	103	0.98 [0.94, 1.00]	0.98 [0.93, 1.00]
Dixit et al. 2018, Dataset 10	49	1	1	34	0.98 [0.89, 1.00]	0.97 [0.85, 1.00]
Dixit et al. 2018, Dataset 12	52	0	3	20	0.95 [0.85, 0.99]	1.00 [0.83, 1.00]
Dixit et al. 2018, Dataset 13	67	0	5	100	0.93 [0.85, 0.98]	1.00 [0.96, 1.00]
Dixit et al. 2018, Dataset 8	176	3	3	85	0.98 [0.95, 1.00]	0.97 [0.90, 0.99]
Dixit et al. 2018, Dataset 9	127	2	2	51	0.98 [0.95, 1.00]	0.96 [0.87, 1.00]
Ibarra-Meneses et al. 2018, Dataset 16	3	0	0	2	1.00 [0.29, 1.00]	1.00 [0.16, 1.00]
Ibarra-Meneses et al. 2018, Dataset 17	3	1	0	1	1.00 [0.29, 1.00]	0.50 [0.01, 0.99]
Itoh and Takagi 2009, Dataset 59	52	0	8	0	0.87 [0.75, 0.94]	Not estimable
Khan et al. 2012, Dataset 42	68	0	7	101	0.91 [0.82, 0.96]	1.00 [0.96, 1.00]
Mukhtar et al. 2018, Dataset 22	82	1	2	100	0.98 [0.92, 1.00]	0.99 [0.95, 1.00]
Mukhtar et al. 2018, Dataset 23	84	1	0	100	1.00 [0.96, 1.00]	0.99 [0.95, 1.00]
Mukhtar et al. 2018, Dataset 24	80	1	4	100	0.95 [0.88, 0.99]	0.99 [0.95, 1.00]
Mukhtar et al. 2018, Dataset 25	82	1	2	100	0.98 [0.92, 1.00]	0.99 [0.95, 1.00]
Sriworarat et al. 2015, Dataset 69	1	0	0	0	1.00 [0.03, 1.00]	Not estimable
Takagi et al. 2009, Dataset 47	8	0	2	0	0.80 [0.44, 0.97]	Not estimable
Verma et al. 2013, Dataset 38	30	18	0	46	1.00 [0.88, 1.00]	0.72 [0.59, 0.82]

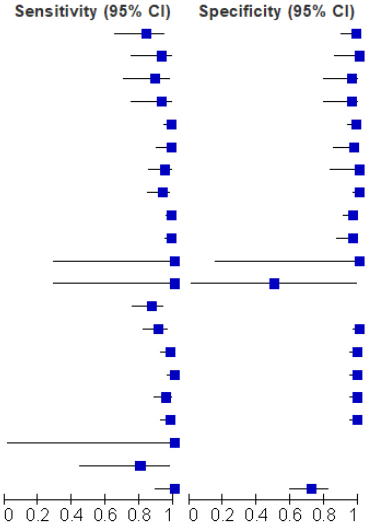

LAMP vs PCR methods for VL diagnosis using whole blood

Study	TP	FP	FN	TN	Sensitivity (95% CI)	Specificity (95% CI)
Abbasi et al. 2016, Dataset 32	3	3	6	32	0.33 [0.07, 0.70]	0.91 [0.77, 0.98]
Abbasi et al. 2016, Dataset 33	7	12	1	23	0.88 [0.47, 1.00]	0.66 [0.48, 0.81]
Abbasi et al. 2016, Dataset 34	5	8	4	27	0.56 [0.21, 0.86]	0.77 [0.60, 0.90]
Adams et al. 2010, Dataset 45	25	1	3	51	0.89 [0.72, 0.98]	0.98 [0.90, 1.00]
de Avelar et al. 2019, Dataset 65	112	2	2	103	0.98 [0.94, 1.00]	0.98 [0.93, 1.00]
Dixit et al. 2018, Dataset 11	15	0	2	80	0.88 [0.64, 0.99]	1.00 [0.95, 1.00]
Dixit et al. 2018, Dataset 13	67	0	5	100	0.93 [0.85, 0.98]	1.00 [0.96, 1.00]
Ghasemian et al. 2014, Dataset 35	42	2	3	30	0.93 [0.82, 0.99]	0.94 [0.79, 0.99]
Ibarra-Meneses et al. 2018, Dataset 20	3	0	0	2	1.00 [0.29, 1.00]	1.00 [0.16, 1.00]
Ibarra-Meneses et al. 2018, Dataset 21	2	1	0	1	1.00 [0.16, 1.00]	0.50 [0.01, 0.99]
Khan et al. 2012, Dataset 43	66	2	6	102	0.92 [0.83, 0.97]	0.98 [0.93, 1.00]
Takagi et al. 2009, Dataset 48	7	1	0	2	1.00 [0.59, 1.00]	0.67 [0.09, 0.99]
Takagi et al. 2009, Dataset 49	8	0	0	2	1.00 [0.63, 1.00]	1.00 [0.16, 1.00]
Thita et al. 2019, Dataset 63	10	0	1	22	0.91 [0.59, 1.00]	1.00 [0.85, 1.00]
Verma et al. 2013, Dataset 36	53	1	2	43	0.96 [0.87, 1.00]	0.98 [0.88, 1.00]
Verma et al. 2017, Dataset 26	64	0	2	76	0.97 [0.89, 1.00]	1.00 [0.95, 1.00]
Verma et al. 2017, Dataset 30	2	0	0	18	1.00 [0.16, 1.00]	1.00 [0.81, 1.00]

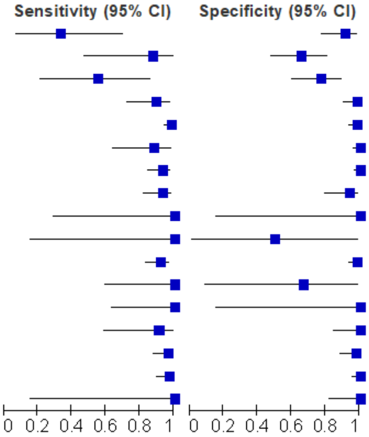

LAMP vs. microscopy for VL diagnosis using other sample types

Study	TP	FP	FN	TN	Sensitivity (95% CI)	Specificity (95% CI)
Ibarra-Meneses et al. 2018, Dataset 14	3	0	0	2	1.00 [0.29, 1.00]	1.00 [0.16, 1.00]
Ibarra-Meneses et al. 2018, Dataset 15	2	1	0	1	1.00 [0.16, 1.00]	0.50 [0.01, 0.99]
Sriworarat et al. 2015, Dataset 70	1	0	0	0	1.00 [0.03, 1.00]	Not estimable
Sriworarat et al. 2015, Dataset 71	1	0	0	0	1.00 [0.03, 1.00]	Not estimable
Verma et al. 2013, Dataset 39	8	7	0	24	1.00 [0.63, 1.00]	0.77 [0.59, 0.90]

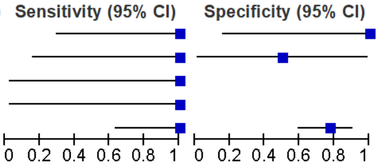

LAMP vs. PCR methods for VL diagnosis using other sample types

Study	TP	FP	FN	TN	Sensitivity (95% CI)	Specificity (95% CI)
Ibarra-Meneses et al. 2018, Dataset 18	3	0	0	2	1.00 [0.29, 1.00]	1.00 [0.16, 1.00]
Ibarra-Meneses et al. 2018, Dataset 19	2	1	0	1	1.00 [0.16, 1.00]	0.50 [0.01, 0.99]
Sriworarat et al. 2015, Dataset 75	1	0	0	0	1.00 [0.03, 1.00]	Not estimable
Sriworarat et al. 2015, Dataset 76	1	0	0	0	1.00 [0.03, 1.00]	Not estimable
Verma et al. 2013, Dataset 37	12	0	3	0	0.80 [0.52, 0.96]	Not estimable
Verma et al. 2017, Dataset 27	15	0	0	0	1.00 [0.78, 1.00]	Not estimable

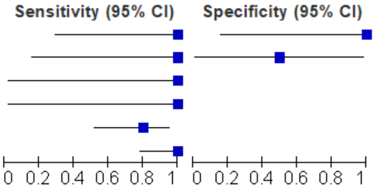

LAMP vs. qPCR for PKDL diagnosis using tissue biopsy

Study	TP	FP	FN	TN	Sensitivity (95% CI)	Specificity (95% CI)
Verma et al. 2013, Dataset 40	60	0	2	24	0.97 [0.89, 1.00]	1.00 [0.86, 1.00]
Verma et al. 2013, Dataset 41	37	22	0	26	1.00 [0.91, 1.00]	0.54 [0.39, 0.69]
Verma et al. 2017, Dataset 28	65	0	2	24	0.97 [0.90, 1.00]	1.00 [0.86, 1.00]
Verma et al. 2017, Dataset 31	2	0	0	19	1.00 [0.16, 1.00]	1.00 [0.82, 1.00]

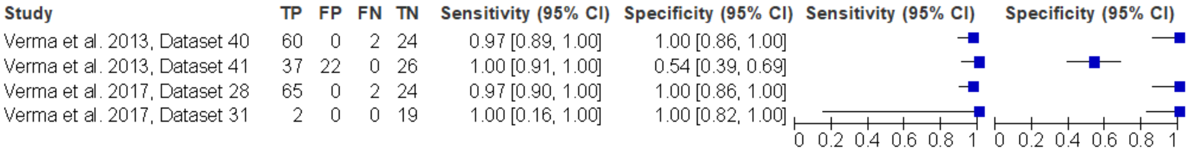

LAMP vs. microscopy for CL diagnosis using skin samples

Study	TP	FP	FN	TN	Sensitivity (95% CI)	Specificity (95% CI)
Chaouch et al. 2019, Dataset 60	37	5	6	24	0.86 [0.72, 0.95]	0.83 [0.64, 0.94]
Imai et al. 2017, Dataset 77	1	0	0	0	1.00 [0.03, 1.00]	Not estimable
Kothawala et al. 2016, Dataset 57	19	0	4	8	0.83 [0.61, 0.95]	1.00 [0.63, 1.00]
Leon et al. 2018, Dataset 6	36	4	0	10	1.00 [0.90, 1.00]	0.71 [0.42, 0.92]
Saki et el. 2019, Dataset 61	75	0	0	0	1.00 [0.95, 1.00]	Not estimable
Schallig et al. 2019, Dataset 52	67	8	12	6	0.85 [0.75, 0.92]	0.43 [0.18, 0.71]
Schallig et al. 2019, Dataset 54	70	7	9	6	0.89 [0.79, 0.95]	0.46 [0.19, 0.75]
Sriworarat et al. 2015, Dataset 66	1	0	1	0	0.50 [0.01, 0.99]	Not estimable
Sriworarat et al. 2015, Dataset 67	2	0	0	0	1.00 [0.16, 1.00]	Not estimable
Sriworarat et al. 2015, Dataset 68	1	0	0	0	1.00 [0.03, 1.00]	Not estimable
Vink et al. 2018, Dataset 50	225	5	12	32	0.95 [0.91, 0.97]	0.86 [0.71, 0.95]
Vink et al. 2018, Dataset 51	237	1	16	20	0.94 [0.90, 0.96]	0.95 [0.76, 1.00]

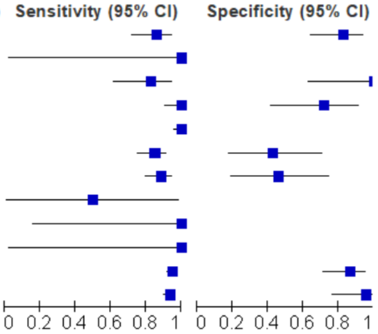

LAMP vs. PCR for CL diagnosis using skin samples

Study	TP	FP	FN	TN	Sensitivity (95% CI)	Specificity (95% CI)
Adams et al. 2010, Dataset 46	42	0	1	0	0.98 [0.88, 1.00]	Not estimable
Chaouch et al. 2019, Dataset 4	42	0	8	22	0.84 [0.71, 0.93]	1.00 [0.85, 1.00]
Chaouch et al. 2019, Dataset 5	27	15	0	30	1.00 [0.87, 1.00]	0.67 [0.51, 0.80]
Imai et al. 2017, Dataset 78	1	0	0	0	1.00 [0.03, 1.00]	Not estimable
Imai et al. 2017, Dataset 79	1	0	0	0	1.00 [0.03, 1.00]	Not estimable
Kothalawala et al. 2016, Dataset 58	19	0	4	8	0.83 [0.61, 0.95]	1.00 [0.63, 1.00]
Leon et al. 2018, Dataset 7	39	1	0	10	1.00 [0.91, 1.00]	0.91 [0.59, 1.00]
Saki et al. 2019, Dataset 62	75	0	0	0	1.00 [0.95, 1.00]	Not estimable
Schallig et al. 2019, Dataset 53	74	1	7	11	0.91 [0.83, 0.96]	0.92 [0.62, 1.00]
Schallig et al. 2019, Dataset 55	77	0	1	14	0.99 [0.93, 1.00]	1.00 [0.77, 1.00]
Sriworarat et al. 2015, Dataset 73	2	0	0	0	1.00 [0.16, 1.00]	Not estimable
Sriworarat et al. 2015, Dataset 74	1	0	0	0	1.00 [0.03, 1.00]	Not estimable
Verma et al. 2017, Dataset 29	8	0	2	24	0.80 [0.44, 0.97]	1.00 [0.86, 1.00]
Vink et al. 2018, Dataset 50	225	5	32	12	0.88 [0.83, 0.91]	0.71 [0.44, 0.90]
Vink et al. 2018, Dataset 51	237	1	20	16	0.92 [0.88, 0.95]	0.94 [0.71, 1.00]

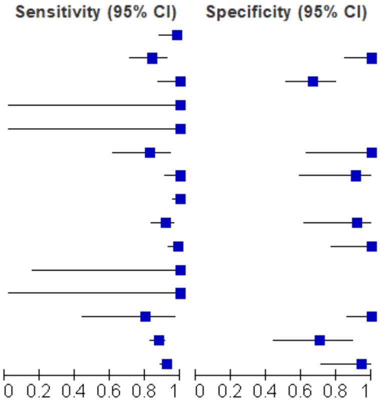

LAMP vs. Microscopy for CL diagnosis using other sample types

Study	TP	FP	FN	TN	Sensitivity (95% CI)	Specificity (95% CI)
Sriworarat et al. 2015, Dataset 66	1	0	1	0	0.50 [0.01, 0.99]	Not estimable
Sriworarat et al. 2015, Dataset 67	2	0	0	0	1.00 [0.16, 1.00]	Not estimable

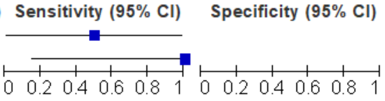

LAMP vs. PCR for CL diagnosis using other sample types

Study	TP	FP	FN	TN	Sensitivity (95% CI)	Specificity (95% CI)
Sriworarat et al. 2015, Dataset 73	2	0	0	0	1.00 [0.16, 1.00]	Not estimable

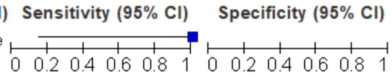

Animal Studies

Study	TP	FP	FN	TN	Sensitivity (95% CI)	Specificity (95% CI)
Alam et al. 2011, Dataset IX	0	0	0	11	Not estimable	1.00 [0.72, 1.00]
Celeste et al. 2019, Dataset I	16	0	2	4	0.89 [0.65, 0.99]	1.00 [0.40, 1.00]
Celeste et al. 2019, Dataset II	7	1	0	1	1.00 [0.59, 1.00]	0.50 [0.01, 0.99]
Chaouch et al. 2013, Dataset V	19	8	16	32	0.54 [0.37, 0.71]	0.80 [0.64, 0.91]
Chaouch et al. 2013, Dataset VI	15	12	5	43	0.75 [0.51, 0.91]	0.78 [0.65, 0.88]
Chaouch et al. 2013, Dataset VII	14	18	5	38	0.74 [0.49, 0.91]	0.68 [0.54, 0.80]
Chaouch et al. 2013, Dataset VIII	23	9	11	30	0.68 [0.49, 0.83]	0.77 [0.61, 0.89]
Gao et al. 2015, Dataset III	12	56	0	43	1.00 [0.74, 1.00]	0.43 [0.33, 0.54]
Gao et al. 2015, Dataset IV	66	3	0	32	1.00 [0.95, 1.00]	0.91 [0.77, 0.98]
Maurelli et al. 2020, Dataset X	24	2	4	30	0.86 [0.67, 0.96]	0.94 [0.79, 0.99]
Maurelli et al. 2020, Dataset XI	0	0	3	57	0.00 [0.00, 0.71]	1.00 [0.94, 1.00]
Maurelli et al. 2020, Dataset XII	10	2	1	47	0.91 [0.59, 1.00]	0.96 [0.86, 1.00]

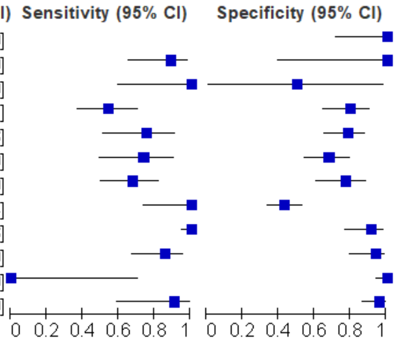

Supplement: Supplementary file 5 — Additional file 5: Figure S2. Forest plots for sensitivity and specificity for all identified datasets. [file 13071_2021_5133_MOESM5_ESM.pdf]
